# Unhealthy Food Television Advertising and Body Weight in Mexican Children

**DOI:** 10.1001/jamanetworkopen.2026.31619

**Published:** 2026-09-01

**Authors:** Alexis Alonso-Bastida, Ana Basto-Abreu, Francisco Reyes-Sánchez, Alan Reyes-García, Martha Carnalla, C. Gabriela García, Lizbeth Tolentino-Mayo, Francesca R. Dillman Carpentier, Juan A. Rivera, Tonatiuh Barrientos-Gutiérrez

**Affiliations:** 1 Centro de Investigación en Salud Poblacional, Instituto Nacional de Salud Pública, Mexico City, México; 2 Centro de Investigación en Nutrición y Salud, Instituto Nacional de Salud Pública, Mexico City, México; 3 Hussman School of Journalism and Media and the Carolina Population Center, The University of North Carolina at Chapel Hill, Chapel Hill

## Abstract

**Importance:**

Exposure to television advertisements for unhealthy food and beverages has been associated with poor dietary habits and childhood obesity. In Mexico, most school-aged children are regularly exposed to advertisements.

**Objective:**

To examine the association of removing television advertisements of unhealthy food and beverages with body weight among school-aged youth.

**Design, Setting, and Participants:**

This decision analytical model used a simulation model based on nonlinear differential equations to estimate 1-year changes in energy intake and obesity by removing television advertisements for unhealthy foods among children and adolescents aged 10 to 17 years. The study used national, representative survey waves from 2022 and 2023 (data collected between September and December of each year) of nonpregnant, nonlactating youths aged 10 to 17 years in Mexico with a plausible body mass index. Data were analyzed from May to December 2025.

**Exposure:**

Complete removal of television advertisements for unhealthy foods and beverages was simulated. For the main scenario, we assumed the intervention would apply to all children and adolescents exposed to television advertising (79.2%) regardless of the time of exposure. Sensitivity analyses varied adherence (30%-100%) and assumed a dose response between exposure time to advertisements and energy intake.

**Main Outcomes and Measures:**

Primary outcomes were changes in daily energy intake, body weight, body mass index, obesity prevalence, and obesity cases.

**Results:**

The analytic sample included 2834 children and adolescents, representing 17 735 608 individuals, with mean television viewing time of 99.8 min/d (95% CI, 92.5-107.2 min/d) and mean daily exposure to unhealthy food advertising of 2.8 min/d (95% CI, 2.6-3.0) min/d. Removing advertisements resulted in a projected mean decrease of 47 kcal/d (95% uncertainty interval [UI], 3-92 kcal/d), leading to a mean weight reduction of 1.2 kg (95% UI, 0.1-2.2 kg) per child in 1 year. Obesity prevalence decreased by 8.3% (95% UI, 0.3%-18.1%), equivalent to 271 000 cases averted. Results assumed no increase in digital marketing.

**Conclusions and Relevance:**

In this decision analytical model, removing unhealthy food and beverage advertisements from television was associated with a substantial reduction in body weight and obesity among children and adolescents in Mexico. These results suggest that strong legislation should be prioritized to protect child health.

## Key Points

**Question:**

Is the removal of television advertising of unhealthy food and beverages associated with a reduction in childhood obesity in Mexico?

**Findings:**

In this decision analytical model using nationwide data from 2022 to 2023 from 2834 children and adolescents, complete removal of television advertising of unhealthy food and beverages was associated with a projected reduction of 47 kcal/d in energy intake and 1.2 kg in body weight per child, corresponding to an 8.3% decrease in obesity prevalence and 271 000 fewer obesity cases in 1 year.

**Meaning:**

This study’s findings suggest that removal of television advertising of unhealthy food may substantially reduce childhood obesity, emphasizing the need for strong legislative action.

## Teaser

This decision analytical model estimates potential changes in energy intake, body weight, and obesity prevalence among Mexican children associated with removal of television advertising of unhealthy food and beverages.

## Introduction

Unhealthy food and beverages are heavily promoted to children and adolescents through television advertisements (ads).^1,2^ Given their poor nutritional quality, they are key drivers of obesity.^3^ Evidence suggests that food ads have a direct impact on children’s dietary preferences, purchase requests, and short-term energy intake.^4^ A meta-analysis^4^ found that exposure to unhealthy food ads, compared with nonfood ads, increases immediate caloric consumption by a mean of 60 kcal and increases preferences for low-nutrient foods.

Mexican children and adolescents are particularly at risk due to high screen time.^5^ In 2022, 82.2% of children (aged 10-14 years) and 90.8% of adolescents (aged 15-19 years) exceeded the recommended 2-hour daily screen limit mainly via television.^5^ This prolonged exposure increases their exposure to unhealthy food ads.^1^ Mexican children were estimated to watch 5.1 food and nonalcoholic beverage ads per hour in 2012 to 2013 on 4 national Mexican broadcast channels, from which 3.9 ads per hour did not meet the World Health Organization (WHO) nutritional composition standards.^6^ In 2023 the WHO called for the mandatory regulation of unhealthy food and beverages marketing directed at children.^7^

In 2014, Mexico restricted the advertising of foods and nonalcoholic beverages to children younger than 12 years on television and in movie theaters. The guidelines set nutritional criteria for products and banned ads for items not meeting these standards during peak child-viewing hours (2:30-7:30 pm on weekdays and 7:00 am-7:30 pm on weekends).^8^ The 2014 guidelines had limitations: (1) lax nutritional criteria; (2) time restrictions that still expose children outside the regulated hours; (3) age restrictions (excluded adolescents); (4) omission of digital media, including internet and social networks; and (5) weak enforcement with no meaningful sanctions.^9^ The guidelines failed to regulate in-program advertising, a loophole exploited by the food industry as seen in 2016 and 2017^10^ as companies placed ads immediately before or after restricted hours, used animated characters, and shifted marketing to less regulated digital platforms. In 2022, amendments to the General Health Law extended the rules to digital media, establishing the same regulations for television, digital, and other media and eliminating the advertising of products with front-of-package warning labels.^11^ However, critics argue that the new regulation is still weak: it reduces the protected hours to only 3 h/d, neglects adolescents, and fails to sufficiently limit persuasive strategies aimed at youths.

In this study, we aimed to estimate the potential change in body weight when removing television ads for unhealthy food and beverages in Mexican school-aged children and adolescents compared with the current state of advertising. We also assessed the potential ramifications of imperfect implementation by ranging adherence from 30% to 100%.

## Methods

**Figure 1.**
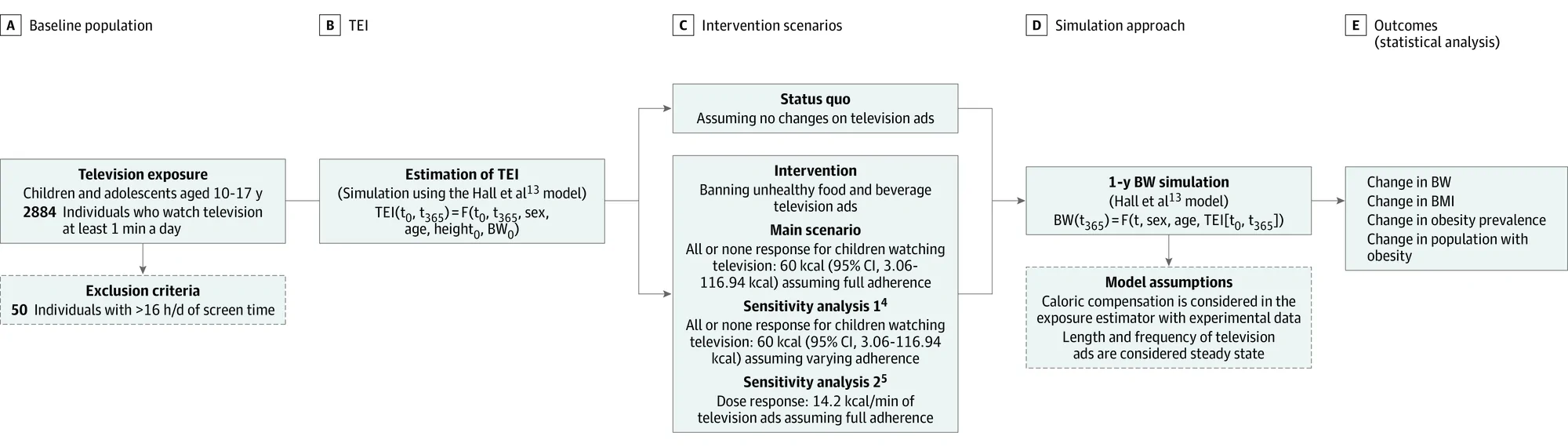
Step-by-Step Diagram of the Modeling Process The study population was composed of children aged 10 to 17 years from the 2022 to 2023 Mexican Health and Nutrition Surveys. Total energy intake (TEI) was estimated assuming no intervention. Four simulations were modeled: (1) status quo, (2) full removal of television advertisements (ads) for unhealthy food and beverages, (3) sensitivity analysis 1, and (4) sensitivity analysis 2. The Hall et al^12^ model for children was used to project body weight (BW) changes during 1 year, accounting for age, sex, growth, and energy intake. Compared with the status quo, each scenario yielded estimates of changes in BW, body mass index (BMI; calculated as weight in kilograms divided by height in meters squared), obesity prevalence, and number of childhood obesity cases.

Figure 1 summarizes the steps of the modeling process. We present the methods divided into 5 subsections: baseline population, total energy intake (TEI), intervention scenarios, simulation approach, and health outcomes (statistical analysis). This study was conducted and reported in accordance with the Consolidated Health Economic Evaluation Reporting Standards (CHEERS) guideline for economic evaluations and decision analytic modeling studies.

### Baseline Population

We used data from the Mexican Health and Nutrition Surveys (ENSANUT) conducted from September to December in 2022 and 2023. Each survey wave is a probabilistic, multistage, stratified, and cluster-sample survey representative of national, regional, rural, and urban populations. In this decision analytical model, we merged 2 subsamples from ENSANUT with data on children and adolescents aged 10 to 17 years: television exposure and anthropometric data, with measurements of body weight and height. The subsample included 2895 children and adolescents. After excluding 11 children with missing or implausible anthropometric data (<−5 or >5 BMI-for-age *z* score) and 50 children with implausible screen time, including television, video games, and computer (>16 h/d), as previously reported.^5^ The final sample had 2834 children, representing 17 735 608 individuals (eAppendix in Supplement 1). This study used secondary data from publicly available sources and was performed considering the ethical principles of the Declaration of Helsinki.^13^ All participants provided written informed consent, and the survey protocol was approved by the Ethics, Research, and Biosafety Committees of the Instituto Nacional de Salud Pública in Mexico.

### Television Exposure

ENSANUT collects mean television screen time on weekdays and weekends. Children were classified as exposed or unexposed to television, with unexposed being children with 0 hours of television exposure weekly, including movies and DVDs. We excluded participants reporting more than 16 hours per day on any screen-based activities (TV, video games, and computers). This threshold was established based on a minimum of 8 hours of sleep for children, aligned with published literature.^5^

### Total Energy Intake

To estimate TEI, we used an equation from Hall et al^12^ that accounts for the daily increases in TEI due to child growth, considering individual’s characteristics, such as sex, age, and baseline height and body weight (more details are available in eAppendix in Supplement 1). This equation (to estimate TEI) was previously calibrated for the Mexican population using 2018 data.^14^ In the current study, we validated the previously calibrated equation for the years 2022 to 2023. An overview of the validation process is presented in eAppendix in Supplement 1. The calibrated TEI equation did not account for external trends in intake, such as increases driven by past obesity trends.

### Intervention Scenarios

For the status quo scenario, we simulated the change in body weight during 1 year considering natural growth under no intervention. For the intervention, we simulated a scenario in which removing unhealthy foods and beverages ads on television would translate into a reduction of daily energy intake among children exposed to television (79.2%), regardless of duration of exposure. The effect size of energy reduction was based on a 2019 meta-analysis by Russell et al,^4^ which included 11 experimental studies, finding that children exposed to food advertising on television consumed a mean increase of of 60.0 kcal/d (95% CI, 3.1-116.9 kcal/d) in energy intake compared with children exposed to nonfood advertising (*I*^2^ = 99.76). The meta-analysis^4^ did not identify a linear association between duration of television exposure and caloric intake, so we assumed an all or none response (ie, children exposed to television had the full caloric effect) (eAppendix in Supplement 1).

### Sensitivities

The caloric effect of removing television ads for unhealthy food and beverages varied by adherence level (sensitivity 1). Imperfect adherence could arise from cross-border advertising (ie, advertising from the US broadcast) or national television channels failing to comply with regulations (ie, incomplete removal of restricted content or nonadherence during certain times of the day). To reflect this possibility, we simulated the impact of implementing the intervention at 30% to 90% adherence in 20% increments.

The dose-response effect (sensitivity 2) assumed a linear association between time exposed to television ads and daily caloric intake reduction. Mytton et al^15^ quantified the dose-response effect as the change in caloric intake per minute of exposure to television ads for unhealthy food and beverages. This parameter was based on 8 studies from the meta-analysis by Russell et al^4^ that reported exposure duration. They estimated that each minute of exposure to television ads was associated with a 14.2-kcal increase in energy intake (95% CI, 0.7-27.7 kcal).^15^ As in the main analysis, we assumed that removing television ads exposure would translate into a corresponding reduction in energy intake.

To estimate exposure time, we first calculated the mean daily time children in Mexico spent watching television by summing reported weekday and weekend screen time and dividing by 7. We then estimated the mean daily exposure to food advertising during TV viewing using the equation: Daily ads exposure_i_ = DailyTvtime_i_(min) × (3.9 Ads/60 min) × (25.9/60 min), where 3.9 ads per hour is the mean advertising frequency for unhealthy food and beverages in Mexico,^6^ 25.9 seconds is the mean duration per ad based on the Nielsen database in UK,^15^ 25 to 30 seconds is the mean length of ads worldwide.^16^*Daily ads exposure_i_* represents the total minutes of unhealthy food and beverages advertising viewed per day by each individual. Under this dose-response scenario, reductions in caloric intake were modeled proportionally to the reduction in advertising exposure time resulting from the intervention.

Using the individual daily advertising exposure in Mexico, we applied the linear effect size proposed by Mytton et al^15^ to estimate the caloric reduction. Additional methodological details are provided in eAppendix in Supplement 1.

### Simulation Approach

We used a microsimulation model proposed by Hall et al^12^ to simulate weight changes in children and adolescents during 1 year. This model uses nonlinear differential equations to capture the dynamics of TEI and natural growth in children and adolescents. It simulates the evolution of factors such as fat mass and fat-free mass composition over time, enabling the estimation of weight changes in individuals aged 5 to 18 years.^12^ Detailed information about the model is available in eAppendix in Supplement 1.

### Statistical Analysis

For all of our scenarios, we projected height for the children and adolescents based on the WHO growth reference.^17^ Outcomes after intervention (body weight, body mass index [BMI; calculated as weight in kilograms divided by height in meters squared]) were calculated using the weight projected through the model of Hall et al^12^ and height estimates based on the WHO reference standards. Obesity prevalence was obtained by classifying individuals into BMI categories using the WHO reference for children and adolescents (overweight: >+1SD *I* score BMI-for-age; obesity: >+2SD *z* score BMI-for-age).^17^ Obesity prevalence was estimated by dividing the obesity cases by the total population. The outcomes were estimated considering the sample design of the ENSANUT. To calculate the CIs, we used the svy function of R, version 4.4.2 (R Foundation for Statistical Computing) for observed data. Data were analyzed from May to December 2025.

We estimated the absolute reductions in body weight, BMI, obesity cases, and obesity prevalence as the difference between the status quo scenario and the intervention scenario after 1 year of simulation. We estimated the relative decrease in body weight, BMI, obesity cases, and obesity prevalence as 1 minus the ratio of means (MR) of the value in the intervention and the status quo multiplied by 100 to express the result as a percentage: [(1 − MR) × 100], where MR indicated intervention/status quo. For the uncertainty intervals, we used 2 primary techniques: bootstrap resampling and Monte Carlo simulation (eAppendix in Supplement 1).

We defined each scenario by key parameters and assumptions (eAppendix in Supplement 1). Baseline population characteristics (weight, height, sex, age, fat mass, and fat-free mass) were incorporated into the model of Hall et al^12^ to simulate weight change. Intervention scenarios included a uniform 60-kcal/d (95% CI, 3.06-116.94 kcal/d) reduction among all individuals exposed to television (all or none response) and a linear reduction of 14.2 kcal/min of advertising exposure (95% CI, 0.7-27.7 kcal/min) (dose response).

## Results

The sample included 2834 participants aged 10 to 17 years, representing 17 735 608 individuals. Among the 17 735 608 individuals, 3 329 831 (18.7%; 95% CI, 15.9%-21.6%) were classified with obesity (3.3 million). Regarding television exposure, 14 051 513 children and adolescents (79.2%; 95% CI, 76.3%-82.2%) were exposed to television, with a mean television viewing time of 99.8 min/d (95% CI, 92.5-107.2 min/d). Mean daily exposure to television ads of unhealthy food was estimated at 2.8 min/d (95% CI, 2.6-3.0 min/d). The characteristics of the study population are presented in eAppendix in Supplement 1.

Table 1 presents simulation results 1 year after the baseline among children and adolescents. Under the status quo, the obesity prevalence would not change significantly with respect to the baseline (18.7% [95% CI, 15.9%-21.6%] vs 18.4% [95% uncertainty interval [UI], 15.4%-21.7%] in the status quo). The main intervention could lead to a mean TEI reduction of 47 kcal (95% UI, 3-92 kcal), resulting in an estimated mean weight reduction of 1.2 kg (95% UI, 2.2-0.1 kg) per person during 1 year. The projected impact on obesity prevalence denotes a relative decrease of 8.3% (95% UI, 18.1%-0.3%) based on a projected status quo prevalence of 18.4% (95% UI, 15.4%-21.7%). This translates into an estimated 272 000 (95% UI, 11 400-581 800) childhood obesity cases averted in 1 year.

**Table 1.** Expected Outcome of Removing Television Advertisements for Nonessential Energy-Dense Food and Sugar-Sweetened Beverages in Mexico^a^

Variable	Total (95% UI)			Relative decrease vs status quo, % (95% CI)^b^^,^^c^
	Status quo	Main intervention	Absolute decrease vs status quo	
Energy intake, kcal/d per person	2407 (2384-2431)	2360 (2310-2412)	47 (3-92)	2.0 (0.1-3.8)
Body weight, kg per person	55.0 (54.1-56.0)	53.8 (52.3-55.3)	1.2 (0.1-2.2)	2.1 (0.1-4.1)
BMI	22.34 (22.00-22.68)	21.86 (21.30-22.45)	0.48 (0.03-0.93)	2.14 (0.12-4.19)
Obesity, pp	18.4 (15.4-21.7)	16.9 (13.4-20.1)	1.5 (0.1-3.3)	8.3 (0.3-18.1)
Obesity cases, No. in thousands	3271 (2704-3918)	2999 (2374-3689)	272 (11-582)	8.3 (0.3-18.1)

Abbreviations: BMI, body mass index (calculated as weight in kilograms divided by height in meters squared); pp, percentage points; UI, uncertainty interval.

^a^ The relative estimation can have minor discrepancies due to rounding.

^b^ The number of decimal places for each outcome was selected based on the variable’s scale and unit of measurement: energy intake and obesity cases (in thousands) are reported with 0 decimal places, body weight and obesity (pp) are presented with 1 decimal place, and BMI is reported with 2 decimal places to reflect the typical precision used for this metric.

^c^ The relative decrease is calculated as 1 – MR (the ratio of means) of the value in the intervention and the status quo multiplied by 100 to express the result as a percentage [(1 − MR) × 100], where MR indicated intervention/status quo.

Table 2 presents the potential outcomes of varying adherence from 30% to 100% of the full effect. A 30% adherence rate could lead to a mean reduction in body weight of 0.3 kg (95% UI, 0.0-0.7 kg) per person. As the intensity of the intervention increases, the reduction in body weight reaches 1.2 kg (95% UI, 0.1-2.2 kg) per person under the full effect scenario. These reductions in weight translate into a relative decrease in childhood obesity prevalence, ranging from 3.5% (95% UI, 0.0%-7.0%) under 30% adherence and 4.8% (95% UI, 0.0%-9.7%) and 6.0% (95% UI, 0.2%-13.7%) under 50% and 70% adherence.

**Table 2.** Expected Outcome of Removing Television Advertisements for Unhealthy Food and Beverages in Mexico Under Different Adherence Levels

Adherence, %	Absolute decrease in body weight (95% UI), kg per person	Relative decrease in obesity (95% UI), %^a^
90	1.0 (0.1-2.0)	7.4 (0.3-16.9)
70	0.8 (0.0-1.6)	6.0 (0.2,13.7)
50	0.6 (0.0-1.1)	4.8 (0.0-9.7)
30	0.3 (0.0-0.7)	3.5 (0.0,7.0)

Abbreviation: UI, uncertainty interval.

^a^ The relative decrease is calculated as 1 – MR (the ratio of means) of the value in the intervention and the status quo multiplied by 100 to express the result as a percentage [(1 − MR) × 100], where MR indicated intervention/status quo.

**Figure 2.**
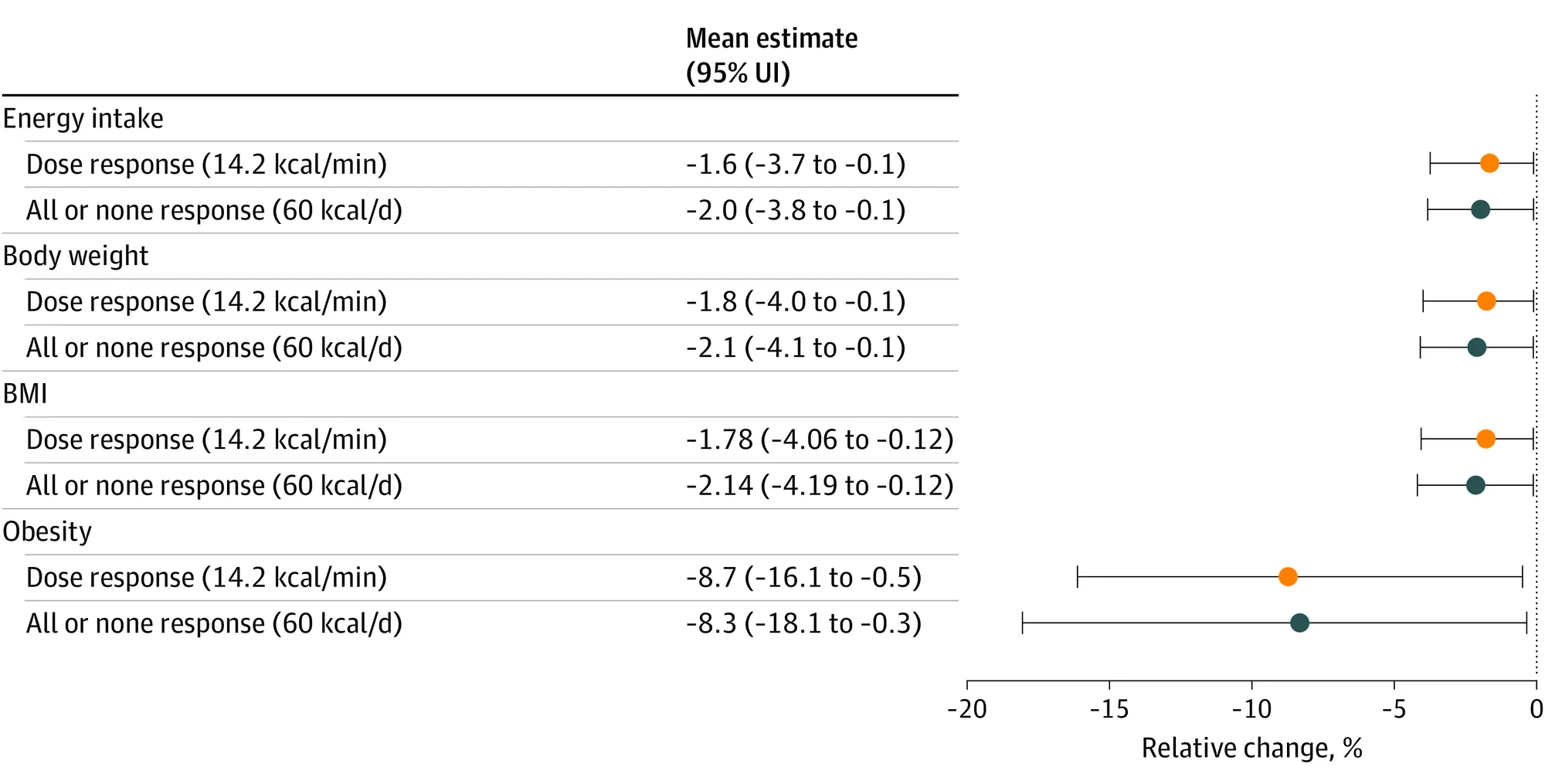
Relative Impact of All or None Response vs Dose Response Modeling Approaches on Obesity Prevalence Dots represent the mean estimates for each outcome, whereas error bars indicate the corresponding 95% uncertainty intervals (UIs). BMI indicates body mass index (calculated as weight in kilograms divided by height in meters squared).

Figure 2 presents the results of simulating the potential outcomes of the intervention assuming dose response compared with the main scenario (all or none). Results for all outcomes were similar.

## Discussion

We aimed to simulate the expected outcome of removing television ads for unhealthy food and beverages on body weight in Mexican school-aged children and adolescents 1 year after implementation. This intervention is projected to decrease energy intake by 47 kcal/d per person, reduce body weight by 1.1 kg per person, and lower obesity prevalence by 1.5 percentage points, representing an 8.3% reduction in obesity. Estimates assume perfect removal of television ads, but adherence is critical: obesity was estimated to decrease 6.0% at 70% adherence and 3.5% at 30% adherence.

Our study projects larger reductions in energy intake (−47 kcal/d) and body weight (1.2 kg) than prior studies.^15,18^ A UK simulation^15^ estimated reductions of −9.1 kcal/d in caloric intake and −0.12 in BMI, and a study^18^ in Australia estimated reductions of −27.5 kcal/d and −0.35, respectively. Differences are not explained by viewing time alone (100 min/d in Mexico, 103 to 120 min/d in the UK, and 71 to 90 min/d in Australia) but by exposure removed. Our study modeled a complete ban across all television programming and hours, equivalent to removing approximately 6.4 unhealthy food ads daily (based on 3.9 ads per hour and 100 min/d of television viewing), whereas the policies modeled in Australia and the UK represented reductions of only 3.6 and 1.5 ads daily, respectively.

Mexico has implemented other structural measures to reduce obesity, including banning sales of unhealthy food and beverages in schools, healthy taxes, and front-of-package warning labels.^19^ We compared our findings with other simulation studies^20,21^ of Mexican children using the same methods (eTable in Supplement 2). The reduction we observed is comparable to banning unhealthy food and beverages in schools, an intervention projected to reduce daily energy intake from 33 to 105 kcal and body weight by 0.8 to 2.5 kg per child during 1 year.^14^ The 8% tax on unhealthy food and 10% tax on unhealthy beverages was expected to have significant but smaller impacts, reducing intake by 17 and 18 kcal/d per person, respectively, and leading to a 0.4-kg reduction in body weight during 1 year.^20,21^ This result aligns with UK evidence, which ranked restricting junk food advertising as one of the most impactful obesity prevention strategies of 33 policies modeled.^22^ Implementing stricter marketing restrictions and school food policies represents an opportunity to shape lifelong dietary behaviors during critical developmental windows.^23^ Early intervention offers advantages over adult interventions because children’s behavioral plasticity allows for more impactful and long-lasting effects.^24^ Removing unhealthy food and beverage ads should be part of Mexico’s comprehensive strategy to curb its metabolic disease epidemic.

Marketing increases the consumption (standardized mean difference, 0.31) and the selection of unhealthy foods (odds ratio, 2.45), with children being at risk due to their susceptibility to brand preference, packaging design, and digital tactics, such as influencers, advergames, and social media.^25,26^ It is urgent to regulate all marketing dimensions (promotion, product, price, and place). Removing television ads for unhealthy food represents a critical step in this direction, but it may also lead to unintended consequences, including the migration of marketing toward unregulated channels, such as online streaming platforms, gaming environments, and other digital spaces where children spend increasing time and are more at risk to subtle, personalized tactics—a shift already occurring globally and further amplified by artificial intelligence and data-driven targeting tools.^27,28,29^ Promotional activity may also intensify in outdoor settings, including vehicle displays, youth-oriented event sponsorships, and locations along school routes, as well as through brand-family advertising of unregulated products or brand-only campaigns that reinforce loyalty and indirectly sustain demand for unhealthy foods.^30,31^ To protect children’s and adolescents’ health, we need to strengthen the current Mexican marketing regulatory law. Although expanding the regulation to digital platforms is a key priority, additional opportunities include prohibiting advertising for brands and products with warning labels, monitor and enforce the law with effective sanctions for nonadherence, and explicitly covering content directed at adolescents not only children in the regulatory framework.^32^

### Limitations

Our study has several limitations. First, it relies on a meta-analytic parameter linking television ad exposure with caloric changes, which may not fully apply to Mexico because most studies included in the meta-analysis^4^ were conducted in high-income countries and focused on younger children (aged 2-12 years), whereas we analyzed children aged 10 to 17 years. Age-related differences and contextual factors may alter susceptibility. Second, the parameter carries uncertainty, limiting stratification by sex, age, or socioeconomic status. Third, the model accounts for growth-related increases in TEI but not obesity-driven trends. Additionally, it only considers television ads, excluding compensatory shifts to digital or outdoor marketing.

## Conclusions

Removing ads for unhealthy food and beverages should be considered a priority in Mexico. Television ads are only a fraction of unhealthy food and beverage ads, yet our decision analytical model suggests that removing television ads is associated with improved children’s health and well-being. Associated outcomes are large and could become a cornerstone of the country’s response to child obesity, adding to fiscal measures, school restrictions, and warning labels. These results provide relevant scientific evidence to guide the formulation of more effective public policies for promoting healthy food environments and defending the rights of children and adolescents in media contexts.

## Notes

Supplement 1. **eAppendix.** Supplementary Methods and Results

Supplement 2. **eTable.** Comparison Between Related Modeling Studies on Changes in Intake, Weight, BMI, and Obesity in Children and Adolescents in Mexico

Supplement 3.
Data Sharing Statement
