# Supplementary material for: Unhealthy Food Television Advertising and Body Weight in Mexican Children
Source: JAMA Netw Open. 2026 Sep 1;9(9):e2631619. doi: 10.1001/jamanetworkopen.2026.31619 (PMC13535789; doi:10.1001/jamanetworkopen.2026.31619)
Supplement: Supplementary file 1 [file jamanetwopen-e2631619-s001.pdf]

Supplemental Online Content

Alonso-Bastida, Basto-Abreu A, Reyes-Sánchez F, et al. Unhealthy food television advertising and body weight in Mexican children. *JAMA Netw Open*. 2026;9(9):e2631619. doi:10.1001/jamanetworkopen.2026.31619

eAppendix. Supplementary Methods and Results

This supplemental material has been provided by the authors to give readers additional information about their work.

© 2026 Alonso-Bastida et al. *JAMA Network Open*.

eAppendix. Supplementary Methods and Results

1 Glossary and Simulation Parameters

Table A presents the glossary of acronyms used throughout the study.

Table A: Glossary of Acronyms Used Throughout the Study

Acronym	*Definition*
CI	Confidence interval.
ENSANUT	Mexican Health and Nutrition Survey (from the Spanish meaning: Encuesta Nacional de Salud y Nutrición).
UI	Uncertainty interval.
WHO	World Health Organization.

Table B presents a summary of the principal simulation parameters used throughout the study.

Table B: Summary of Model Symbols and Definitions

Symbol	*Definition*
I_{ref}	Intake reference.
E_{ref}	Reference energy expenditure.
EB_{ref}	Reference energy balance.
K	Constant for the baseline energy expenditure.
Γ	Regression coefficients describing the relationship between resting metabolic rate and fat mass or fat-free mass.
FM	Fat mass.
FFM	Fat-Free mass.
Δ	Physical activity function.
H	Biochemical coefficient associated with fat and protein synthesis.
P	Energy densities.
P	Energy partitioning ratio.
BMI	Body mass index.
BW	Body weight.
Age	Age.
TEI	Total energy intake.
T	Simulation time point.
kcal reduction(d)	Kilocalorie reduction per day in each intervention.

© 2026 Alonso-Bastida et al. *JAMA Network Open*.

2 Data sources

We used anthropometric data of individuals aged 10-17 years old from the Mexican Health and Nutrition Survey (ENSANUT, from its Spanish acronym) 2022-23. Individuals aged 10-17 were taken as the baseline sample (initial sample) for simulation. More details about the anthropometric data are presented in the next subsection.

2.1 Anthropometric data

We obtained weight and height measurements from 14,160 individuals aged 10–17 years using standardized procedures. Measurements were converted to Z scores using the 2006 WHO reference [12, 10]. We excluded 11 individuals with missing data or extreme values for height-for-age (< −6 or > +6 Z score) and BMI-for-age (< −5 or > +5 Z score) [12, 10]. Following ENSANUT 2018 criteria, we excluded those with implausible BMI values: children (6–11 years) with BMI < 10 or > 38kg/m^2, adolescents (12–18 years) with BMI < 10 or >58kg/m^2, and pregnant/lactating adolescents. We also excluded 50 individuals with implausible data in screen time. The final analytic sample included 2,834 individuals, representing an expanded data set of 17,735,608 individuals. Data processing is summarized in Figure A.

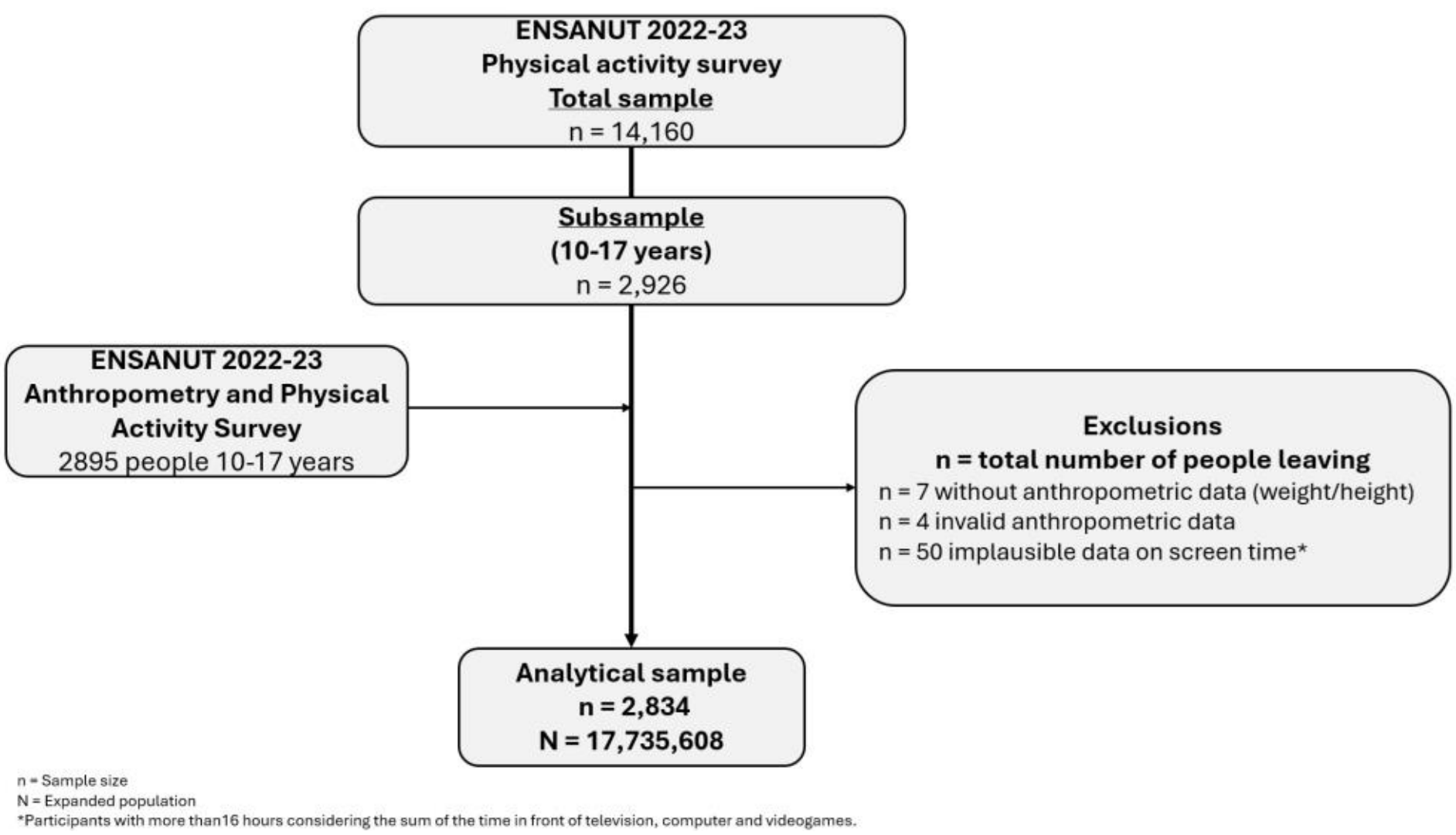

Fig A: Data processing

© 2026 Alonso-Bastida et al. *JAMA Network Open*.

2.2 Screen time in children and adolescents

We quantified the screen time of Mexican children and adolescents (ages 10-17) using the anthropometry and physical activity databases from the Mexican Health and Nutrition Survey (ENSANUT, from its Spanish acronym) 2022-23. The physical activity section of ENSANUT includes specific questions regarding screen exposure time.

- **Television:** 1) On a weekday, how many hours Monday through Friday do you spend in front of a screen, watching television (including movies or DVDs)? Include morning, afternoon and evening time; 2) Similar question differentiated by "on a weekend day".
- **Video games:** 1) On a weekday, how many hours from Monday to Friday do you spend in front of a screen, playing video games (Atari, Sega, Nintendo, Game Boy, PlayStation, Wii, Xbox, or other video and/or computer games, electronic tablet or phone), include morning, afternoon and evening time; 2) Similar questioning differentiated by "on a weekend day".
- **Computer, tablet or phone:** 1) On a weekday, how many hours from Monday to Friday do you spend in front of a screen, either chatting, surfing the internet, sending mails or doing homework (not counting playing on the computer, tablet or phone), include morning, afternoon and evening time; 2) Similar questioning differentiated by "on a weekend day".

For each individual, we calculated the average screen time by summing usage over 5 weekdays and 2 weekend days. The total was then divided by 7 to obtain the weekly average for each item. Following [7], individuals who spent more than 16 hours per day on screen-based activities, such as television viewing, video games, and computer use, were excluded. Our focus was specifically on television screen time, considering the enforcement of current Mexican regulations on the advertising for unhealthy food and beverages.

2.3 Data sources linking caloric intake and exposure to unhealthy food and beverages advertising

As the primary source for our study on exposure to unhealthy food ads on television and its relationship with increased caloric intake, we used the meta-

© 2026 Alonso-Bastida et al. *JAMA Network Open*.

analysis conducted by Russell et al. [11]. This analysis includes 12 experimental studies that evaluated the association between exposure to food advertising on the screen and dietary intake among children aged 2 to 18 years. The results indicate an average increase of 60 kcal/day (95% CI: 3.06, 116.94) attributable to exposure to television advertising.

3 Total energy intake by day

We used the methodology presented by Hall et al. [4] to estimate the baseline daily total energy intake for each individual in the sample:

$$I_{ref}(t) = E_{ref}(t) + EB_{ref}(t); \quad (1)$$

where $I_{ref}(t)$ represents the reference energy intake, which corresponds to the energy required for normal growth in children, assuming no intervention. We define $E_{ref}(t)$ as the reference energy expenditure and $EB_{ref}(t)$ as the reference energy balance, computed as $EB_{ref}(t) = I_{ref}(t) - E_{ref}(t)$.

3.1 Reference energy balance

To estimate the reference energy balance (EB_{ref}), we used an equation adapted from Katan et al. [4, 5]:

$$EB_{ref}(t) = A_{EB} \cdot e^{-\frac{t-t_A^{EB}}{\tau_A^{EB}}} + B_{EB} \cdot e^{-\frac{\left(t-t_B^{EB}\right)^2}{2\left(\tau_B^{EB}\right)^2}} + D_{EB} \cdot e^{-\frac{\left(t-t_D^{EB}\right)^2}{2\left(\tau_D^{EB}\right)^2}}. \quad (2)$$

The parameters and corresponding values used in (2), as defined by Katan et al., are summarized in Table C.

Table C: Parameters for the energy balance equation adapted from Katan el al. [5]

Parameter	Males	Females
A_{EB}	7.2	16.5
B_{EB}	30.0	47.0
D_{EB}	21.0	41.0
τ_A^{EB}	15.0	7.0
τ_B^{EB}	1.5	1.0
τ_D^{EB}	2.0	1.5
t_A^{EB}	5.6	4.8

© 2026 Alonso-Bastida et al. *JAMA Network Open*.

t_B^{EB}	9.8	9.1
t_D^{EB}	15.0	13.5

3.2 Energy expenditure of reference

To estimate the reference energy expenditure, we used the equation proposed by Hall et al. [4], shown in (3):

$$E_{ref}(t) = K + (\gamma_{FFM} + \delta(t))FFM_{ref}(t) + (\gamma_{FM} + \delta(t))FM_{ref}(t) + \frac{\eta_{FFM}}{\rho_{FFM}}(p \cdot EB_{ref}(t) + g(t)) + \frac{\eta_{FM}}{\rho_{FM}}((1-p) \cdot EB_{ref}(t) - g(t)) \quad (3)$$

In this formulation, K is a sex-specific constant representing baseline energy expenditure ($K = 800$ kcal/d for males, $K = 700$ kcal/d for females). The parameters $\gamma_{FM} = 4.5$ kcal/kg/d and $\gamma_{FFM} = 22.4$ kcal/kg/d are regression coefficients describing the relationship between resting metabolic rate and fat mass (FM) or fat-free mass (FFM), respectively [9]. The variables $FM_{ref}(t)$ and $FFM_{ref}(t)$ represent reference fat mass and fat-free mass at time t, based on age, sex, and BMI category-specific median values (see [1] for details). Parameters $\eta_{FM} = 180$ kcal/kg and $\eta_{FFM} = 230$ kcal/kg account for the biochemical coefficients associated with fat and protein synthesis. The corresponding energy densities are $\rho_{FM} = 9400$ kcal/kg and $\rho_{FFM} = 4.3 \cdot FFM_{ref}(t) + 837$ kcal/kg. Finally, p denotes the energy partitioning ratio, i.e., the proportion of energy from $EB_{ref}(t)$ allocated to fat-free mass, estimated using the Forbes equation (4). [3]

$$p = \frac{C}{C + FM_{ref}(t)} \quad (4)$$

where:

$$C = 10.4 \times \frac{\rho_{FFM}}{\rho_{FM}}. \quad (5)$$

The physical activity function, δ, included in (3) is defined as follows (6):

$$\delta(t) = \delta_{min} + \frac{(\delta_{max} - \delta_{min})P^h}{t^h + P^h}. \quad (6)$$

The minimum physical activity level across all ages and sexes is represented by the constant $\delta_{min} = 10$ kcal/kg/d. In contrast, the maximum physical activity level is sex-specific: $\delta_{max} = 19$ kcal/kg/d for males and $\delta_{max} = 17$ kcal/kg/d for females. The parameter $P = 12$ years denotes the age at which physical activity peaks, while the constant $h = 10$ governs the rate of its subsequent decline with age.

© 2026 Alonso-Bastida et al. *JAMA Network Open*.

The function $g(t)$ in (3) accounts for the energy cost of children's body growth. It is estimated using the following expression (7), adapted from Hall et al. and Katan et al. [4, 5]:

$$g(t) = A \cdot e^{-\frac{t-t_A}{\tau_A}} + B \cdot e^{-\frac{(t-t_B)^2}{2\tau_B^2}} + D \cdot e^{-\frac{(t-t_D)^2}{2\tau_D^2}}; \quad (7)$$

Table D summarizes the sex-specific parameters used in the $g(t)$ function.

Table D: Parameters for the growth function $g(t)$

Parameter	Males	Females
A	3.2	2.3
B	9.6	8.4
D	10.1	1.1
τ_A	2.5	1.0
τ_B	1.0	0.9
τ_D	1.5	0.7
t_A	4.7	4.5
t_B	12.5	11.7
t_D	15.0	16.2

3.2.1 Reference for fat-free mass and fat mass

Briefly, we described the process of estimation followed by Hall et al. [4]. The daily reference values for fat mass ($FM_{ref}(t)$, Equation 8) and fat-free mass ($FFM_{ref}(t)$, Equation 9) were estimated at the population level using linear interpolation. To achieve this, the authors first calculated the mean fat mass and fat-free mass by age and sex. Next, individuals were classified into four BMI categories: Underweight, Normal weight, Overweight, and obesity. BMI was categorized in underweight, normal, overweight, and obesity based on the WHO's growth reference pattern for children and adolescents [10]. Finally, we applied the equations developed by Deurenberg et al. [2] to estimate individual fat mass and fat-free mass values within the sample.

$$FM = \begin{cases} \frac{1.51 \cdot \text{BMI} - 0.7 \cdot \text{age} - 2.2}{100} \times \text{BW} & \text{if male} \\ \frac{1.51 \cdot \text{BMI} - 0.7 \cdot \text{age} + 1.4}{100} \times \text{BW} & \text{if female} \end{cases} \quad (8)$$

© 2026 Alonso-Bastida et al. *JAMA Network Open*.

$$FFM = BW - FM \quad (9)$$

where BMI and BW stand for body mass index and body weight, respectively.

4 Intervention scenarios

We analyzed the impact of removing the television ads for unhealthy food and beverages through four weight change simulation scenarios targeting the Mexican population aged 10 to 17 years:

4.1 Status Quo

This scenario assumes no external changes in individual caloric intake. Caloric consumption is modeled based solely on the natural progression associated with age-related growth, without any intervention-driven reductions.

4.2 Main scenario, All or None Response (60.00 kcal per day)

This scenario applies to the original estimate from Russell et al. [11]. It assumes a direct reduction of 60 kcal/day (95% CI: 3.06, 116.94) in TEI among children and adolescents who watch television, following the removal of all unhealthy food and beverage ads. The intervention is restricted to individuals reporting regular television viewing.

4.3 Sensitivity analysis, Dose-response caloric reduction (14.20 kcal per minute)

In sensitivity analysis, we considered a linear dose–response relationship between exposure time to unhealthy food and beverages ads and associated caloric reduction (14.2 kcal/min (95% CI: 0.7, 27.7)), based on the methodology outlined by Mytton et al. [8].

We estimated the average time each individual spends watching unhealthy food and beverages ads based on the following assumptions:

- Unhealthy food and beverages ads appear at an average frequency of 3.9 advertisements per hour. [6]

© 2026 Alonso-Bastida et al. *JAMA Network Open*.

- Each advertisement has an average duration of 25.9 seconds. [8]

We estimated the average calorie intake attributable to this advertising based on the following assumption:

- There is a linear dose-response relationship between advertising exposure time and calorie consumption, with an estimated rate of 14.2 kcal/min (95% CI: 0.7, 27.7). [8]

5 Micro-simulation model

We simulated the change in body weight for each individual j in the dataset using (10):

$$BW_j(t_{365}) = BW_j^{model}(age_j + t_{365}; sex_j, FFM(t_0), FM(t_0), TEI(t_0, t_{365})), \quad (10)$$

In (10), $t_0 = 0$ denotes the initial time point (corresponding to the year 2025), and $t_{365} = 365$ indicates 1 year of simulation, expressed in days. The terms $FFM(t_0)$ and $FM(t_0)$ represent the initial fat-free mass and fat mass, respectively, for each child or adolescent, estimated using the Deurenberg equations (9 and 8). The variable $TEI(t_0, t_{365})$ is a vector of daily total energy intake from $t_0 = 0$ to day $t_{365} = 365$, determined according to the specific scenarios considered in the analysis. For the status quo scenario, $TEI(t)$ is defined as shown in (11).

$$TEI(t_0, t_{365}) = \left(I_{ref}(0), I_{ref}(2), \ldots, I_{ref}(d), \ldots, I_{ref}(365)\right), \quad (11)$$

$I_{ref}(0)$ and $I_{ref}(d)$ denote the reference energy intake at the initial time and on day d, respectively, as estimated in Section 3. For the intervention scenarios, the daily energy intake on day d was calculated by applying the corresponding kilocalorie reduction (12):

$$I_{scenario}(d) = I_{ref}(d) - kcal\ reduction(d), \quad (12)$$

where $\text{kcal reduction}(d)$ represents the reduction in kilocalories applied each day under the intervention scenarios. The magnitude of this reduction depends on the type of intervention implemented; the specific reductions associated with each intervention are detailed in Section 4. Consequently, in the intervention scenarios, total energy intake over the simulation period is determined by the cumulative application of (13).

© 2026 Alonso-Bastida et al. *JAMA Network Open*.

$$TEI(t_0, t_{365}) = \left(I_{scenario}(0), I_{scenario}(1), \ldots, I_{scenario}(d), \ldots, I_{scenario}(365)\right). \quad (13)$$

5.1 Hall's model

Briefly, the Hall model [4] is a dynamic model designed to describe changes in body weight among children and adolescents aged 5 to 17 years. Based on the principle of energy balance, considering both energy expenditure and total energy intake for each individual, the model simulates the temporal evolution of body weight, fat-free mass, and fat mass. The core equations underlying this model are presented in Equations (14) to (18).

$$EB(t) = I(t) + E(t); \quad (14)$$

$$E(t) = K + \gamma_{FFM}FFM + \gamma_{FM}FM + \beta\Delta I(t) + \delta\, BW(t) + \eta_{FFM}\frac{dFFM}{dt} + \eta_{FM}\frac{dFM}{dt}; \quad (15)$$

$$BW(t) = FFM(t) + FM(t); \quad (16)$$

$$\frac{dFFM}{dt} = \frac{p(I-E)+g}{\rho_{FFM}}; \quad (17)$$

$$\frac{dFM}{dt} = \frac{(1-p)(I-E)-g}{\rho_{FM}} \quad (18)$$

In this study, we assume no trend in energy intake over time ($\Delta I = 0$), given that the simulation spans a 1-year period. Under this assumption, the reference energy intake (i.e., in the absence of any intervention) is estimated as follows (19):

$$I_{ref} = EB_{ref} + E_{ref} \quad (19)$$

where:

$$\begin{aligned} I_{ref} &= EB_{ref} + E_{ref} \quad (20)\\ &= EB_{ref} + K + \gamma_{FFM}FFM_{ref} + \gamma_{FM}FM_{ref} + \delta BW_{ref} + \eta_{FFM}\frac{dFFM}{dt} + \eta_{FM}\frac{dFM}{dt} \\ &= EB_{ref} + K + \gamma_{FFM}FFM_{ref} + \gamma_{FM}FM_{ref} + \delta BW_{ref} \\ &\quad + \frac{\eta_{FFM}\left(p\left(EB_{ref}+g\right)\right)}{\hat{\rho}_{ref}} + \eta_{FM}\frac{(1-p)\left(EB_{ref}\right)-g}{\rho_{FM}} \\ &= EB_{ref} + K + \left(\gamma_{FFM}+\delta\right)FFM_{ref} + \left(\gamma_{FM}+\delta\right)FM_{ref} \end{aligned}$$

© 2026 Alonso-Bastida et al. *JAMA Network Open*.

$$+\frac{\eta_{FFM}}{\hat{\rho}_{FFM}}\left(pEB_{ref}\ +\ g\right)+\frac{\eta_{FM}}{\rho_{FM}}\left(\left(1-p\right)EB_{ref}-g\right)$$

A complete description of the model structure and its associated parameters can be found in Hall et al. [4].

5.2 Simulation pseudocode

Fig. B illustrates the pseudocode of the simulation process for the body weight change model proposed by Hall et al. [4].

Algorithm 1: Pseudocode for the Body Weight Change Model.

Begin

Clean the environment

Load required libraries:

- '*bw*' (to use Hall model)
- '*anthroplus*' (to apply the WHO 2007 growth reference for children and adolescents)

Data preparation:

- *Read ENSANUT 2022-2023 database*
- Extract relevant variables: sex, weight, height, age, BMI, screen time, and survey weights
- Filter dataset based on inclusion criteria.

BMI categorization:

- Use WHO growth standards via the '*anthroplus*' package to classify BMI.

Total energy intake

- Estimate individual baseline energy requirements.
- Apply calorie reduction according to the intervention scenario.

Simulate body weight change

For i = 1 to N (number of individuals in the sample)

• Simulate weight and BMI changes over one year using Hall's model

end

Post-simulation analysis:

• *Calculate mean values and confidence intervals for:*

- *Energy intake*
- *Body weight*
- *BMI*
- *Obesity prevalence*
- *Estimated number of obesity cases*

End

Fig B: Pseudocode of the simulation process for Hall et al.'s Body Weight Change Model.

© 2026 Alonso-Bastida et al. *JAMA Network Open*.

6 Uncertainty intervals

To estimate uncertainty intervals for each projection scenario of body weight among Mexican children and adolescents aged 10 to 17, we first assessed the uncertainty surrounding the average body weight. A replicated bootstrap approach based on the Rao-Wu-Yue-Beaumont method was implemented, generating 1,000 replicate samples. Replicate weights were derived from the original complex survey design using the bootstrap design function. For each bootstrap iteration, weighted means were computed, and the empirical distribution of these means was used to construct percentile-based uncertainty intervals. Specifically, the 2.5th and 97.5th percentiles were extracted to approximate the 95% confidence interval. These bootstrap-derived intervals were compared to standard design-based confidence intervals estimated via "svymean()" and "confint()", enabling an inferential contrast between both approaches.

Based on the 1,000 bootstrap-generated replicate designs and the uncertainty reported for each intervention scenario (all or none reduction of 60.00 kcal/day [95% CI: 3.06, 116.94]; dose-response reduction of 14.4 kcal/day [95% CI: 0.70, 27.70]), a Monte Carlo analysis was conducted. For each scenario, 1,000 random values of caloric intake reduction were sampled from a normal distribution constrained by the respective confidence intervals reported in the literature. The Hall model for body weight change over 1 year was then applied to each simulated dataset. From the resulting distributions, we computed 95% confidence intervals for energy intake, body weight, body mass index (BMI), obesity prevalence, and the estimated number of obesity cases using the 2.5th and 97.5th percentiles. Table E presents the parameters and assumption used to re-estimate the projection of body weight.

All statistical analyses and simulations were performed using R version 4.4.2 (R Foundation for Statistical Computing), incorporating the survey and "svrep" packages for complex survey design and bootstrap resampling, respectively. Data were analyzed from May to December 2025.

© 2026 Alonso-Bastida et al. *JAMA Network Open*.

Table E: Parameter definitions, value ranges, data sources, and uncertainty modeling assumptions.

Data	Description	Value (95% CI)	Data source	Accounted uncertainty	Assumptions
Model structure	Validated dynamic simulation model of children, that predicts the time course of individual weight change in response to eliminate television ads in Mexico.<br>This model considers dynamic physiological adaptations that occur with body weight changes at individual level and considers changes in children's growth.	Not applicable	Hall et al. [4]	Described in the following rows	Status quo scenario assumed body weight as steady state, implying that no external factors (e.g., dietary changes, exercise programs, or medical treatments) alter weight over time. The model does not account for trends in caloric consumption.
Model parameters					
Baseline					
Weight	Measured body weight (kg)	Average: 52.8 (51.7, 53.9)	ENSANUT 2022–2023	Sampling design. Accounted using bootstrap	Not applicable
Height	Measured height (m)	Average: 1.54 (1.53, 1.55)	ENSANUT 2022–2023	Sampling design. Accounted using bootstrap	Not applicable
Sex	Individual's reported sex	*Female %:* 48.1 (44.3, 52.0)<br>*Male %:* 51.9 (48.0, 55.7)	ENSANUT 2022–2023	Sampling design. Accounted using bootstrap	Not applicable
Age	Individual's reported age in years	Average: 13.8 (13.6, 13.9)	ENSANUT 2022–2023	Sampling design. Accounted using bootstrap	Not applicable
Body fat mass (FM)	Fat mass (kg) estimated for each individual.	Eq. (8)	Deurenberg et al. [2]	Sampling design. Accounted using bootstrap	Not measured
Body fat-free mass (FFM)	Fat-free mass (kg) estimated for each individual.	Eq. (9)	Deurenberg et al. [2]	Sampling design. Accounted using bootstrap	Not measured

© 2026 Alonso-Bastida et al. *JAMA Network Open*.

Status quo (No intervention)					
Total energy intake (TEI) by day	Vector of daily intake for each individual (kcal/day)	Eq. (11)	Hall et al. [4]	Monte Carlo. The parameter value was randomly selected assuming a normal distribution, using its mean and standard deviation	Not measured
Intervention					
Effect: All-or-none response	Uniform reduction due to television exposure	-60.00 kcal/day (3.06, 116.94)	Russell et al. [11]	Monte Carlo. The parameter value was randomly selected assuming a normal distribution, using its mean and standard deviation.	Everyone exposed to television experiences a uniform reduction of 60 kcal/day in total energy intake.<br>We assumed no changes in digital marketing of unhealthy food and beverages.
Effect: Dose-response	Linear reduction by television ad time	-14.20 kcal/min (0.70, 27.70)	Mytton et al. [8]	Monte Carlo. The parameter value was randomly selected assuming a normal distribution, using its mean and standard deviation.	Caloric reduction is modeled as a linear function of junk food television advertisement exposure time.
Law compliance	Reduction under compliance law	30% to 100% kcal/day	Proposed in this work	Not applicable	The effect of compliance is modeled at 30% to 100% of the main intervention.

Abbreviations: ENSANUT : National Health and Nutrition Survey

© 2026 Alonso-Bastida et al. *JAMA Network Open*.

7 Model calibration

The original Hall's body weight model was previously calibrated for the 2018 Mexican children and adolescents by Basto-Abreu et, al. [1]. We validated this calibration for the data used in our analysis. We compared simulated average body weights of children aged 10-17, with observed average body weights of children 11-18 from the ENSANUT 2022-23 (Figure C). The simulated body weights were obtained from 1 year of simulation without intervention. Our 1-year predictions were consistent with the observed average weights by nutritional status for the corresponding ages in the ENSANUT survey, with an average error of 1.9 kg.

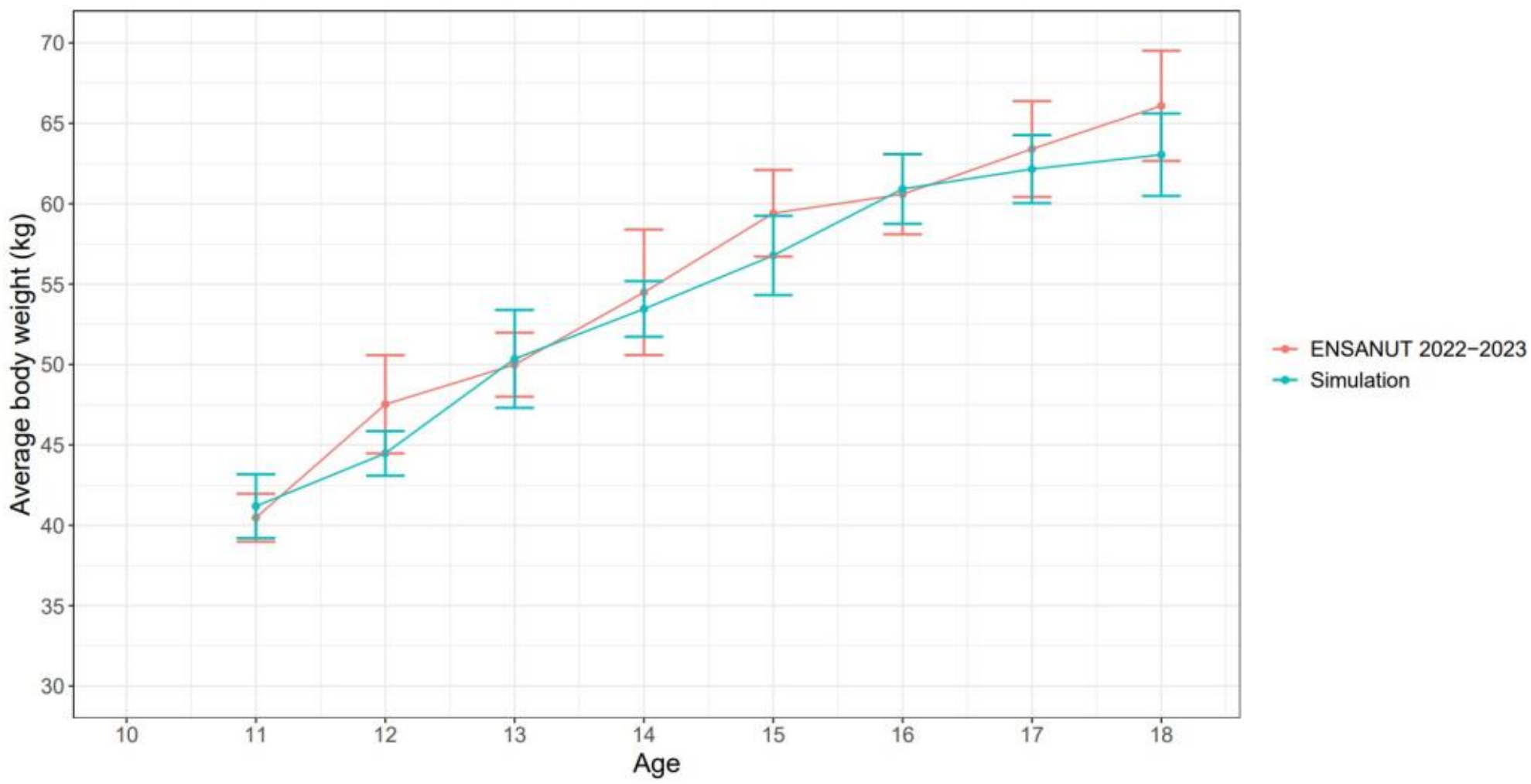

Fig C: Comparison of mean body weight of children aged 10 to 17 years old between the Dynamics of Childhood Growth and Obesity Model and mean observed values from the National Health and Nutrition Survey 2022-2023 (ENSANUT 2022-2023)

8 Results

This section presents the characteristics of the study population and other simulation results: 1) the main analysis already presented in the main manuscript, but with more decimal digits; 2) the sensitivity analysis, assuming a doses response between time of TV ads of unhealthy foods and caloric reduction. Each intervention scenario is compared with the status quo, highlighting key outcomes: energy intake, body weight,

© 2026 Alonso-Bastida et al. *JAMA Network Open*.

BMI, childhood obesity prevalence, and the number of obesity cases. We report both absolute values and relative changes based on projections after 1 year, using the status quo as the reference.

8.1 Characteristics of the study population

Table F presents the population characteristics of the sample analyzed. The mean age of the population was 13.2 years (95% CI: 13.0, 13.5). Women comprised 47.9% of the sample (95% CI: 44.0, 51.8). The majority of participants resided in urban areas, accounting for 73.9% (95% CI: 68.9, 78.4). Most of the population lived in the central geographic region, representing 47.5% (95% CI: 42.6, 52.5). Socioeconomic status (SES) was distributed as follows: 38.7% (95% CI: 34.6, 42.9) in the low SES category, 28.4% (95% CI: 25.6, 31.4) in the middle SES category, and 32.9% (95% CI: 29.0, 37.0) in the high SES category.

Table F: Characteristics of the study population, 10-17 years at baseline. Mexico, ENSANUT 2022-2023

Population characteristics		Mean or proportion	95% CI
**Age**		13.2	(13.0, 13.5)
**Sex**			
	Women	47.9	(44.0, 51.8)
	Men	52.1	(48.2, 56.0)
**Urbanity**			
	Rural	26.1	(21.6, 31.1)
	Urban	73.9	(68.9, 78.4)
**Geographic region**			
	North	19.9	(15.1, 25.8)
	Center	47.5	(42.6, 52.5)
	South	32.5	(28.5, 36.8)
**SES**			
	Low	38.7	(34.6, 42.9)
	Middle	28.4	(25.6, 31.4)
	High	32.9	(29.0, 37.0)

Abbreviations: 95% CI: Confidence interval. SES: Socioeconomic status.

© 2026 Alonso-Bastida et al. *JAMA Network Open*.

8.2 Main scenario: All or None Response (60.00 kcal per day)

Table G shows the simulation results after 1 year for both the Status quo scenario and the main intervention scenario, which assumes a fixed reduction of 60.00 kcal/day (95% CI: 3.06, 116.94). Among children and adolescents, this intervention leads to an average daily energy intake reduction of 47 kcal (95% UI: 3, 92), resulting in an estimated average weight decrease of 1.16 kg per person (95% UI: 0.07, 2.24) over 1 year. The projected impact on obesity prevalence indicates a relative reduction of 8.32% (95% UI: 0.35, 18.06), based on a projected status quo prevalence of 18.44% (95% UI: 15.44, 21.65). This change translates into an estimated 271.97 thousand (95% UI: 11.35, 581.79) childhood obesity cases averted over the course of 1 year.

Table G: Results of the main analysis: All or none response (60.00 kcal per day)

	Status Quo	All or none response	Absolute decrease vs. Status Quo	Relative decrease vs. Status quo
	Total (95% UI)	Total (95% UI)	Total (95% UI)	% (95% UI)
Energy intake, kcal/d per person	2407 (2384, 2431)	2360 (2310, 2412)	47 (3, 92)	1.97 (0.11, 3.83)
Body weight, kg per person	54.96 (54.07, 55.90)	53.80 (52.35, 55.33)	1.16 (0.07, 2.24)	2.10 (0.12, 4.09)
BMI	22.34 (22.00, 22.68)	21.86 (21.30, 22.45)	0.48 (0.03, 0.93)	2.14 (0.12, 4.19)
Obesity, pp	18.44 (15.44, 21.65)	16.91 (13.36, 20.13)	1.53 (0.06, 3.29)	8.32 (0.35, 18.06)
Obesity cases, No. in thousands	3270.58 (2704.25, 3918.10)	2998.61 (2374.24, 3689.02)	271.97 (11.35, 581.79)	8.32 (0.35, 18.06)

Abbreviations: BMI, body mass index (calculated as weight in kilograms divided by height in meters squared); pp, percentage points; UI, uncertainty interval.

8.3 Sensitivity analysis: Dose-response caloric reduction (14.20 kcal per minute)

Table H presents the simulation results of the sensitivity analysis for the dose response scenario. In this scenario, we considered a reduction of 14.20 kcal/min (95% CI: 0.70, 27.70) in exposure to unhealthy food and beverages television ads. This reduction translated into a decrease of 40 kcal per person per day (95% UI: 3, 90) in total energy intake compared to the status quo scenario. The caloric decrease resulted in a relative body weight reduction of 1.75% (95% UI: 0.11, 3.99), corresponding to an average BMI reduction of 0.40 kg/m^2 per person (95% UI: 0.03, 0.91). This intervention was associated with the prevention of approximately 271.72 thousand cases of childhood obesity (95% UI: 17.72, 525.23), representing an 8.46% (95% UI: 0.50, 16.13) relative reduction in obesity prevalence compared to the projected prevalence after 1 year in the status quo scenario.

© 2026 Alonso-Bastida et al. *JAMA Network Open*.

Table H: Results of the sensitivity analysis: Dose-response caloric reduction (14.20 kcal per minute)

	Status Quo	Dose response	Absolute decrease vs. Status Quo	Relative decrease vs. Status quo
	Total (95% UI)	Total (95% UI)	Total (95% UI)	% (95% UI)
Energy intake, kcal/d per person	2407 (2384, 2431)	2367 (2312, 2413)	40 (3, 90)	1.65 (0.11, 3.74)
Body weight, kg per person	54.96 (54.07, 55.90)	53.99 (52.43, 55.35)	0.97 (0.06, 2.20)	1.76 (0.11, 3.99)
BMI	22.34 (22.00, 22.68)	21.94 (21.33, 22.45)	0.40 (0.03, 0.91)	1.78 (0.12, 4.06)
Obesity, pp	18.44 (15.44, 21.65)	16.83 (13.67, 20.06)	1.61 (0.10, 2.93)	8.73 (0.50, 16.13)
Obesity cases, No. in thousands	3270.58 (2704.25, 3918.10)	2985.06 (2363.49, 3557.48)	285.52 (17.72, 525.23)	8.73 (0.50, 16.13)

Abbreviations: BMI, body mass index (calculated as weight in kilograms divided by height in meters squared); pp, percentage points; UI, uncertainty interval.

© 2026 Alonso-Bastida et al. *JAMA Network Open*.

References

[1] Ana Basto-Abreu et al. “Predicted impact of banning nonessential, energydense food and beverages in schools in Mexico: A microsimulation study”. In: *PLoS Medicine* 21.5 (2024), e1004394.

[2] Paul Deurenberg, Jan A Weststrate, and Jaap C Seidell. “Body mass index as a measure of body fatness: age-and sex-specific prediction formulas”. In: *British journal of nutrition* 65.2 (1991), pp. 105–114.

[3] Gilbert B Forbes. “Lean body mass-body fat interrelationships in humans.” In: (1987).

[4] Kevin D Hall et al. “Dynamics of childhood growth and obesity: development and validation of a quantitative mathematical model”. In: *The lancet Diabetes & endocrinology* 1.2 (2013), pp. 97–105.

[5] Martijn B Katan et al. “Impact of masked replacement of sugar-sweetened with sugar-free beverages on body weight increases with initial BMI: secondary analysis of data from an 18 month double–blind trial in children”. In: *PLoS One* 11.7 (2016), e0159771.

[6] Bridget Kelly et al. “Global benchmarking of children’s exposure to television advertising of unhealthy foods and beverages across 22 countries”. In: *Obesity Reviews* 20 (2019), pp. 116–128.

[7] Catalina Medina et al. “Prevalencia de comportamientos del movimiento en poblaci´on mexicana”. In: *salud pu´blica de m´exico* 65 (2023), s259–s267.

[8] Oliver T Mytton et al. “The potential health impact of restricting lesshealthy food and beverage advertising on UK television between 05.30 and 21.00 hours: a modelling study”. In: *PLoS medicine* 17.10 (2020), e1003212.

[9] Karl M Nelson et al. “Prediction of resting energy expenditure from fatfree mass and fat mass”. In: *The American journal of clinical nutrition* 56.5 (1992), pp. 848–856.

[10] Mercedes de Onis et al. “Development of a WHO growth reference for school-aged children and adolescents”. In: *Bulletin of the World Health Organization* 85.9 (Sept. 2007), pp. 660–667. issn: 0042-9686. doi: 10.2471/BLT.07.043497. url: https://www.who.int/bulletin/ volumes/85/9/07-043497/en/.

© 2026 Alonso-Bastida et al. *JAMA Network Open*.

[11] Simon J Russell, Helen Croker, and Russell M Viner. "The effect of screen advertising on children's dietary intake: A systematic review and metaanalysis". In: *Obesity reviews* 20.4 (2019), pp. 554–568.

[12] WHO Multicentre Growth Reference Study Group. *WHO Child Growth Standards: Length/Height-for-Age, Weight-for-Age, Weight-for-Length, Weight-for-Height and Body Mass Index-for-Age: Methods and Development*. Available from: https://www.who.int/publications/i/item/924154693X. Geneva: World Health Organization, 2006. url: https://www.who.int/publications/i/item/924154693X.

© 2026 Alonso-Bastida et al. *JAMA Network Open*.
