# Supplementary material for: Unhealthy Food Television Advertising and Body Weight in Mexican Children
Source: JAMA Netw Open. 2026 Sep 1;9(9):e2631619. doi: 10.1001/jamanetworkopen.2026.31619 (PMC13535789; doi:10.1001/jamanetworkopen.2026.31619)
Supplement: Supplementary file 2 [file jamanetwopen-e2631619-s003.pdf]

Data Sharing Statement

Alonso-Bastida. Unhealthy Food Television Advertising and Body Weight in Mexican Children. *JAMA Netw Open*. Published September 01, 2026. doi:10.1001/jamanetworkopen.2026.31619

Data
Data available: Yes
Data types: Deidentified participant data
How to access data: The dataset will be made available at a public repository URL once the peer-review process has been completed, allowing us to incorporate any required revisions beforehand.
When available: With publication

Supporting Documents
Document types: Statistical/analytic code
How to access documents: The analytic code will be made available at a public repository URL once the peer-review process has been completed, allowing us to incorporate any required revisions beforehand.
When available: With publication

Additional Information
Who can access the data: anyone
Types of analyses: The data will be made available for research purposes only, including replication of the published findings and secondary analyses consistent with the objectives of the original study.
Mechanisms of data availability: The data will be made available through a public repository after publication without investigator support.
